# How Chemical and Sensorial Markers Reflect Gentian Geographic Origin in Chardonnay Wine Macerated with *Gentiana lutea* Roots?

**DOI:** 10.3390/foods9081061

**Published:** 2020-08-05

**Authors:** Manon Biehlmann, Samvel Nazaryan, Emily Krauss, Mike Iron Ardeza, Stéphanie Flahaut, Gilles Figueredo, Jordi Ballester, Céline Lafarge, Elias Bou-Maroun, Christian Coelho

**Affiliations:** 1 Master 2 Microbiology and Physicochemistry of Food and Wine Processes, Agrosup Dijon, Université de Bourgogne Franche Comté, 21000 Dijon, France; manon.biehlmann@agrosupdijon.fr (M.B.); samvel.nazaryan@agrosupdijon.fr (S.N.); emily.krauss@agrosupdijon.fr (E.K.); iron-mike.ardeza@agrosupdijon.fr (M.I.A.); 2 CPPARM, ZA Les Quintrands, Route de Volx, 04100 Manosque, France; stephanie.flahaut@cpparm.org; 3 LEXVA Analytique, 7 rue Henri Mondor, Biopole Clermont Limagne, 63360 Saint Beauzire, France; g.figueredo@lexva-analytique.com; 4 Centre des Sciences du Goût et de l’Alimentation, AgroSup Dijon, CNRS, INRA, Univ. Bourgogne Franche-Comté, F-21000 Dijon, France; jordi.ballester@u-bourgogne.fr; 5 PAM UMR A 02.102 PAM, AgroSup Dijon, Université Bourgogne Franche Comté, AgroSup Dijon, PAM UMR A 02.102 PAM, CEDEX 21078 Dijon, France; celine.lafarge@agrosupdijon.fr (C.L.); elias.bou-maroun@agrosupdijon.fr (E.B.-M.)

**Keywords:** macerated wine, *Gentiana lutea*, bitter taste, secoiridoids, traceability

## Abstract

A Burgundian Chardonnay wine was enriched with *Gentiana lutea* root powders originating from two French mountain sites (Massif Central and Jura) in order to prepare semi-dry gentian aromatized Chardonnay wine-based drinks. These novel alcoholic beverages were chemically and sensorially characterized for evaluating if the gentian geographic origin influenced bitter and elemental and volatile composition and sensory profiles in the final products. For that, the chemical fingerprint of gentian powders and wines were carried by headspace solid phase microextraction gas chromatography coupled to mass spectrometry (HS–SPME–GC), liquid chromatography coupled to diode array detector (LC–DAD) and inductive coupled plasma optical emission spectroscopy (ICP–OES). The mineral and volatile analysis show that the geographic distinction is more obvious in gentian powders compared to gentian macerated wines. Interestingly the maceration process in Chardonnay wine involves extraction processes revealing statistical distinctions in other chemical markers of gentian origin, like for amarogentin and loganic acid or some mineral elements such as barium and aluminum that affect undoubtedly bitterness perception and sensory properties in macerated wines compared to unmacerated wine. Additionally, the gentian volatile 2-methoxy-3-sec-butylpyrazine and the Chardonnay wine volatile ethyl-9-decenoate differentiated, respectively by extraction and powder adsorption mechanisms could be responsible of more subtle sensory differentiations between macerated wines from two distinct gentian origins.

## 1. Introduction

Bitterness in wine is generally attributed to flavonoids and non-flavonoids compounds originating from grapes or ellagitannins conferred during wood aging [1,2,3,4]. Some microbiologic deviations appear to generate acrolein responsible of bitterness development in wines [5]. Bitterness increases with alcoholic strength and is modulated by sugar content and pH [6]. Even if winemaking procedures could confer higher amounts of phenols and tannins, the bitter taste perceived in wine generally decreases with aging time due to polymerization reactions of these bitter compounds [7,8]. Usually, persistent bitterness in wine should be avoided [9]. However, in some other beverages, bitterness is desired for example in the case of macerated wine products, vermouth wines or bitter spirits [4,10].

Bitterness perception mechanism has been proven to be different among consumers and its sensorial evaluation is temporally dynamic due to its perception mechanism [9,11]. Bitterness sensation has been ascribed to complex combined mechanisms of transduction, i.e., the activation of taste cells located at the root of the tongue and generation of intracellular signals caused by taste stimuli [12]. On one hand, bitterness taste stimuli can be directly provoked on apical ion channels by compounds like quinine sulfate or some divalent cations like Ca^2+^, Mg^2+^, Mn^2+^. The latter inhibit K^+^ conductance process and therefore increase the depolarization process required for action potential elicitation for bitterness perception. In the opposite, K^+^ or Na^+^ ions suppress bitterness perception by facilitating K^+^ conductance in taste cells. On the other hand, a receptor-mediated transduction mechanism has been proposed for bitterness perception for compounds in alcoholic beverages such as condensed tannins, polyphenols, isohumulones, amino acids requiring cellular enzymes like phospholipase C or phosphodiesterase for the depolarization process [4,13].

Among natural plants conferring bitter properties, *Gentiana lutea* has been well described and used for decades in the food industry due to its high concentration of secoiridoids and xanthones [14,15,16,17]. Among these compounds, amarogentin (secoiridoid glycoside) is the most bitter compound. Two other glycosides, gentiopicroside and loganic acid, are the most abundant bitter compounds found in gentian roots [16,18]. Additionally, these compounds possess health benefits and could be used for pharmaceutical purposes [19,20,21,22,23,24]. Nevertheless, *Gentiana lutea* use should be managed sustainably by maintaining the resource, promoting new products and authenticating its chemical composition.

Volatile compounds from *Gentiana lutea* are responsible of gentian flavor typicity, which are associated with diverse chemical families such as aldehydes, terpenoids, alcohols and pyrazines conferring herbal and green characters [25]. Other ketones, fatty acids and benzenes derivatives volatile compounds were found in gentian roots [26]. The volatile composition of gentian roots was reliably explored by an headspace solid phase microextraction (HS-SPME) coupled to gas chromatography–mass spectrometry (GC–MS) and was able to discriminate between different commercial and cultivated/wild gentian roots [26].

In this study, gentian roots powders originating from two French mountain sites (Massif Central and Jura) and their macerates in Chardonnay wines, were analyzed in terms of bitter compounds, mineral and volatile composition with the objectives: (i) to determine if chemical markers could reflect the geographic origin of gentian in the macerated wines and (ii) to assess bitterness and to describe the aroma of the macerated wines compared to the control wine and (iii) to discuss the potential discriminations related to gentian origin in terms of chemical and sensory properties in the resulting gentian macerated wines.

## 2. Materials and Methods

### 2.1. Study Design

*Gentiana lutea* roots were sampled entirely in July 2018 from two sites: Chapelle des Bois in Jura (J) and Picherande in Massif Central (MC). Three gentian roots per site, presenting the same physiological stage C [27] were harvested to take in account the variability of the site. All roots were cleaned from their residual earth, manually sliced in 1–2 cm pieces, dried at 40 °C for two days and finally ground to a fine powder and stored at 4 °C. Six gentian root powders J1, J2, J3, MC1, MC2 and MC3 were chemically analyzed. For bitter and mineral composition, gentian roots were extracted at (weight/volume) ratio of 10 g/1 L of methanol. For volatile composition, dry powder was used in state.

The same solid to liquid ratio was used for preparation of gentian macerated wines, as pointed out in traditional recipes [28]. Ten g of each gentian powders were macerated in 1 L of a Chardonnay white wine elaborated in September 2019 at Domaine Expérimental de l’Université de Bourgogne, with an automatic shaker at 100 rpm (Infors AG CH-4103, Bottmingen, Switzerland) for one month at 20 °C. A control wine (CW) was exposed under the same conditions as for those used for gentian macerated wines without the maceration step. The seven wine samples labelled J1W, J2W, J3W, MC1W, MC2W, MC3W and CW were filtrated in 1 µm Merck Millipore (Burlington, NJ, USA) before being chemically analyzed in technical triplicates, referenced a, b and c.

### 2.2. Analytical Methods

#### 2.2.1. Bitter Content

Amarogentin, gentiopicroside and loganic acid, supplied by Extrasynthèse (Genay, France) at the highest purity grade, were dissolved in methanol at 1 g/L for stock solution preparation, stored at 4 °C. Bitter compounds were analyzed by liquid chromatography using a ultra-high performance liquid chromatography coupled to a photodiode array detector (Acquity, Waters) device equipped with a Raptor ARC C18 column (150 × 2.1 mm, particle size of 1.8 µm) and a mobile phase made of (ultrapure water/acetonitrile/formic acid) with (84.9/15/0.1), *v/v*/*v*). Volume injection was set at 1 µL. Loganic acid, gentiopicroside and amarogentin were, respectively detected at 260, 280 and 305 nm. Retention times of pure standards were 6.0, 7.1 and 9.4 min for loganic acid, gentiopicroside and amarogentin, respectively. UV spectra comparisons with pure standards were used for identification. Quantification was validated by an external calibration from 1 to 100 mg L^−1^ and standard addition of the mix of commercial standards from 1 to 100 mg L^−1^ to gentian methanolic and wine extracts. Analysis were performed in technical triplicates.

#### 2.2.2. Mineral Composition

Al, As, B, Ba, Ca, Cd, Co, Cu, Fe, K, Mg, Mn, Na, Sr and Zn pure standards were purchased from Inorganic Ventures (Christiansburg, VA, USA). Calibration solutions were prepared in 10% ethanol from 0.01 to 0.1 mg·L^−1^ for As, Cd, Co, Mn, Sr, Zn; from 0.5 to 5 mg·L^−1^ for Al, B, Ba, Cu, Fe; from 10 to 100 mg·L^−1^ for Ca, Mg, Na and from 100 to 1000 mg·L^−1^ for K. Measurements were performed with an inductively coupled plasma optical emission spectrometer PlasmaQuant PQ 9000 Elite from Analytic Jena (Jena, Germany). Operational conditions were set as follows: coolant gas flow rate at 15 L·min^−1^, carrier gas flow rate at 0.65 L·min^−1^, auxiliary gas flow rate at 0.65 L·min^−1^, RF generator power at 1400 W, integration time of 0.5 to 5 s. All gentian root powders methanolic extracts and gentian macerated wines were acidified with HNO_3_ 1% and injected with a flow rate at 1 mL·min^−1^ and a quartz torch injector kit to enhance nebulization for alcoholic solutions. Analysis were performed in technical triplicates.

#### 2.2.3. Analysis of Volatile Compounds by HS–SPME–GCMS

The extraction of volatile compounds was done for all the different samples, by headspace solid-phase microextraction and analyzed by GC–MS (HS–SPME–GCMS).

This analysis was carried out using a three-phase fiber (divinylbenzene (DVB)/Carboxen (CAR)/polydimethylsiloxane (PDMS), 50/30 µm, Supelco). Before use, the fiber was conditioned in accordance with the manufacturer’s recommendatios. In order to optimize the extraction, a preliminary study was done to choose the best extraction parameters (choice of fiber, extraction parameters). The selected extraction conditions were the following:

For each gentian powder: 1 g of powder in a 20 mL vial were incubated in a water bath at 40 °C for 15 min. Then the fiber was exposed to the sample headspace for 15 min and was desorbed for 10 min into GC–MS. The analyses were done in technical triplicate;

For each gentian macerated wines and the control wine: 10 mL of wine in a 20 mL vial were incubated in a water bath at 40 °C for 15 min. Then the fiber was exposed to the sample headspace for 30 min and was desorbed for 10 min into GC–MS. The analyses were done in technical triplicate.

Volatiles were analyzed with a mass spectrometer (Agilent 5975C-VLMSD, electronic impact at 70 eV) paired with an Agilent 7890A gas chromatograph fitted with a split/splitless injector (240 °C). The chromatograph was equipped with a capillary column DBWAX of 30 m × 0.32 mm (J&W Scientific). The film thickness was 0.25 µm. Helium was used as carrier gas at a rate of 1.5 mL·min^−1^ (average velocity of 44 cm·s^−1^). The temperature of the oven was increased from 40 °C to 240 °C at 4 °C·min^−1^ and maintained 5 min at 240 °C. The injection temperature was 240 °C and was done in spitless mode.

The mass spectrometer was used in scan mode from *m/z* 29 to 400. The corresponding volatile compounds were tentatively identified by matching their spectral fragmentation with those provided by the mass spectral library of the National Institute of Standards and Technology (NIST) and the Wiley Registry (WILEY).

In addition, for each volatile compound obtained, linear retention index (LRI) was calculated using the retention times of a standard mixture of C7–C30 saturated alkanes (Sigma-Aldrich) and compared with the LRI values published in the literature for columns with the same polarity.

In order to identify robust gentian marker, volatile compounds systematically present at least in two of the three technical replicates and in the three biologic replicates for at least one of the two geographic sites have been kept. If a compound was absent in one of the three biologic triplicates by geographic site, it was eliminated for the statistical treatment.

#### 2.2.4. Gentian Macerated Wines Enological Parameters

Classical enological parameters: ethanol (%), total acidity (g·L^−1^ tartaric acid), malic acid (g·L^−1^), glucose (g·L^−1^), pH and volatile acidity (g·L^−1^ acetic acid) of the gentian macerated wines were measured by FT-IR spectroscopy (OenoFossTM analyzer).

Additionally, wine color was estimated by visible absorption in the 380–740 nm with a CM-5 Konica Minolta spectrophotometer, using optical glass precision cells of 50 mm pathlength (Hellma analytics). By using illuminant D65 and a standard observer at 10° of visual field, L* (lightness from 0 to 100) a* (redness from −100 to +100) and b* (yellowness from −100 to +100) values were attributed to each wine.

### 2.3. Sensory Analysis

Replicates of the macerated wine samples within the same geographic site were pooled into one sample for sensory analysis. The two macerated samples, coded JW (Jura) and MCW (Massif Central), were then adjusted with 80 g/L of food-grade powdered sucrose (Beghin Say) in order to obtain semi-dry aromatized Chardonnay wines. The control wine coded CW had no sucrose addition.

Sensory sessions were carried out by a panel of 30 enology students (15 males and 15 females average age 22 years old) in a sensory room equipped with individual booths at 21 °C. The 25 mL samples were presented in dark ISO wine glasses. The samples were presented monadically and the order of presentations was balanced across tasters. Tasters were asked to assess each sample from the left to the right and rate the bitter intensity on an eleven points structured scale anchored from zero (absence of bitterness) to 10 (very intense bitterness). Right after the bitterness rating, they were asked to freely describe the samples in terms of odor/aroma.

Prior to the session a reference standard made of a quinine sulfate solution at 20 mg/L was presented to illustrate the high anchor of the bitterness scale.

### 2.4. Statistical Analysis

Statistical data analysis was carried out using R studio software. Student’s *t*-test and analysis of variance (ANOVA) was performed on the values of bitter compounds and mineral concentration obtained from the gentian roots and macerated wine analysis, based on 9 values per geographic site (3 technical triplicates × 3 biologic triplicates).

Principal component analysis and Hierarchical cluster analysis were done with the software Perseus 1.5.1.6 (Max Planck Institute of Biochemistry, Martinsried, Germany). The exploration of the volatile dataset on gentian roots and gentian macerated wines was based on two criteria: (i) minimum of 8 numeric values on the 9 available for a geographic site and (ii) a statistical difference between sites with a *p*-value below 0.05. The selection of valid volatile markers in macerated wines was completed with an additional validation by working on areas obtained on the extracted ions chromatograms specific to the major fragments of each molecular features.

Concerning sensory analysis, bitterness scores distributions were tested for normality using Shapiro–Wilk test. Since data distributions significantly deviated from normality for all three samples, sensory data were analyzed using non-parametric Friedman test followed by Nemenyi post hoc test. The free vocabulary description was first lemmatized, then the terms related to the same or close sensations were pooled together. For instance, vegetal, green, herbaceous and green bell pepper citations were added into a “global” descriptor, namely vegetal. Then the descriptors with less than 6 (20% of the panel) citations at least for one of the samples were removed from the dataset. When a panelist suggested two specific attributes with close meaning or belonging to the same category only one citation was taken into account, in order not to overestimate the citations of the global descriptor. The resulting “samples × descriptors” frequency table was submitted to correspondence analysis (CA). All the sensory data were analyzed with XLSTAT 2020 (Addinsoft, Paris, France).

## 3. Results

### 3.1. Chemical Markers of Gentian Root Origin Present in Gentian Root Powders

Chemical analysis resulting from gentian root powders were carried with the objectives to identify molecular chemical markers enabling to differentiate geographic origin of gentian harvest that could influence sensorial properties of macerated beverages. Mean concentrations of bitter compounds and minerals are presented in Table 1. They reveal that bitter compounds (gentiopicroside, amarogentin, loganic acid) are present in the same order of magnitude in weigh percentage wt % (7–7.25%, 0.02–0.03%, 0.98–1.06%, respectively) as pointed out in previous studies [16,18] but they do not permit to differentiate the two sampling sites since the values are statistically not different (*p*-value > 0.05). Interestingly root mineral content were also very similar between the two geographic sites for the following elements Al, As, B, Ba, Cd, Co, Cu, Fe, K, Mn, Na, Sr and Zn, but only differ for Ca and Mg. Compared to MC, J gentian roots contain approximatively seven times more calcium and approximatively 2.7 times less magnesium. Such discrepancies could be related to differences in soil elements assimilation by gentian in the two sampling sites and should be deepened in futures studies [29].

**Figure 1:**
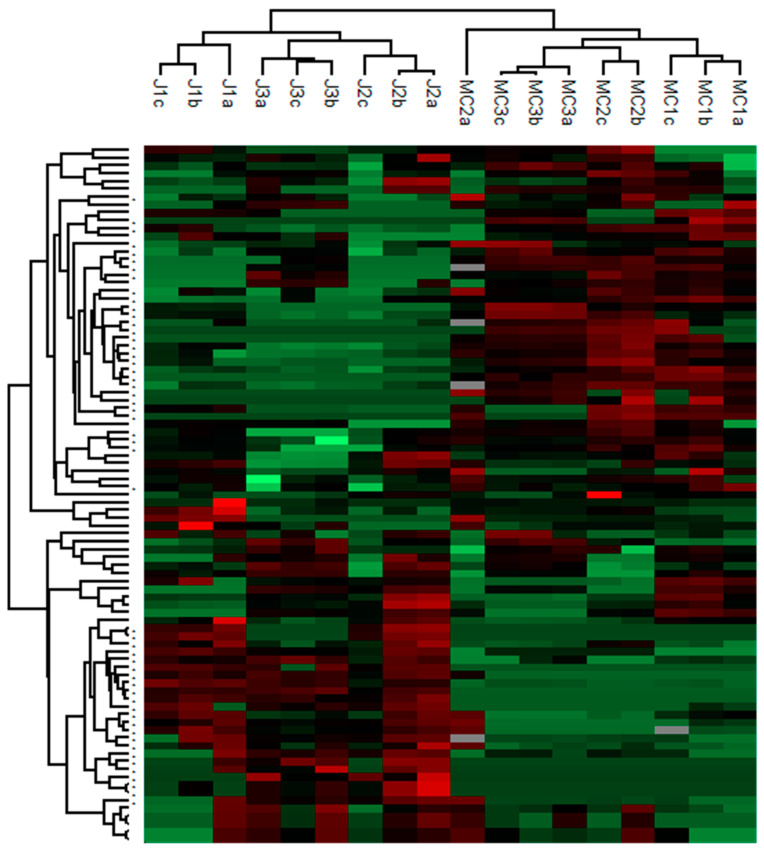
Hierarchical cluster analysis (HCA) performed on all normalized volatile compound areas in gentian roots from the two sampling sites.

Non-volatile compounds were assessed in this study with an unsupervised approach in order to isolate chemical markers of geographic origin. As shown in Figure 1, the heatmap representation and the hierarchical cluster analysis highlight that MC and J gentian roots were fairly discriminated by applying strict analytical and statistical criteria based on geographic considerations. Among the 186 detected volatile compounds in the gentian root chromatograms, 30 were specific to MC, 22 were specific to J and 27 were common to both sites. They presented a statistical difference between geographic sites (*p*-value < 0.05). Some of them were already described in previous studies [25,26]. As the objective of this study was to evaluate their presence in gentian macerated wines, the identification step of all these gentian origin markers should be detailed in an upcoming study. Nevertheless, among the volatiles presenting the highest relative areas, we found that MC gentian amounts of limonene, eucalyptol and benzene-1-methyl-2-(1-methyl ethyl) were significantly higher compared to J gentian roots. Others like trans-β-ocimene or 1,3,5,7-cyclooctatetraene were specific to MC gentian root due to its total absence in J gentian roots. Among the common volatiles: nonanal, estragole, 1,2-dimethoxybenzene, linalool, trans-pinocarveol, methyl salicylate, already described in gentian roots [26] and present in all our samples did not permit the differentiation of the two sites.

Analysis were performed in technical triplicates (a,b,c). The color spans from green (minimum normalized area) to red (maximum normalized area) detected for each volatile compound. The symbol + (on the left) indicates a significant difference (*p*-value < 0.05) between the two geographic sites.

### 3.2. Chemical Markers of Gentian Root Origin Present in Gentian Macerated Wines

Table 2 indicate the classical oenological parameters of Chardonnay white wines macerated with MC and J gentian roots. Even if evident differences could be diagnosed between non macerated wines and macerated wines, no statistical differences between all these enological classical parameters enable to discriminate the macerated wines MCW and JW (*p*-value > 0.05).

Nevertheless Table 2 interestingly reflects that root powder extraction in Chardonnay white wine by the maceration process tend to differentiate geographic sites with amarogentin and loganic acid (gentiopicroside is still not statistically different between the two sites). These results could be ascribed to a higher solubility of amarogentin compared to gentiopicroside in ethanol/water–solution [30]. When looking to the mineral content of macerated wines, calcium is still differentiating JW with higher mean values (114.49 mg·L^−1^) compared to MCW (84.46 mg·L^−1^), but the order of difference has changed from 7 in powder methanolic extracts compared to 1.3 in the resulting wines. Magnesium is even no more statistically differing from MC to J due to the elevated content of Mg element in the unmacerated wine, reaching up to 80 mg·L^−1^ (Table S1). Interestingly, other mineral elements such as Sr, Al, K and Ba mean concentrations were statistically differentiating the two gentian sampling sites.

**Figure 2:**
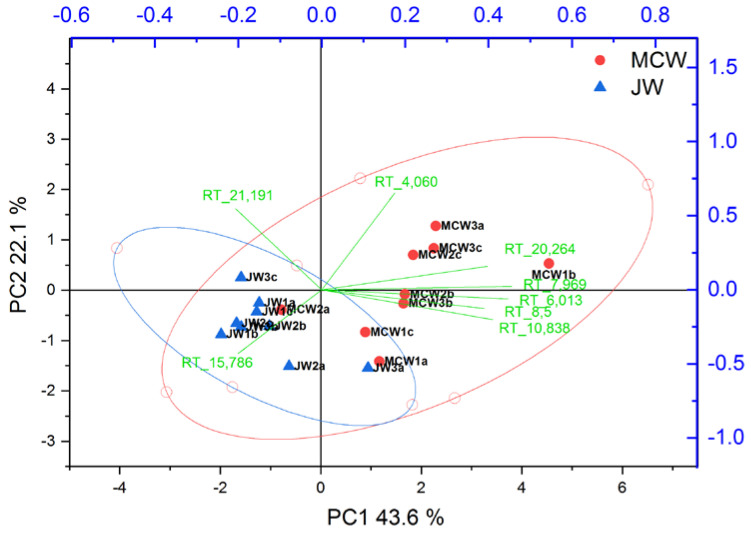
Principal component analysis (PCA) of macerated wines performed on the area of 8 volatile constituents statistically different from the two sites based on ANOVA and *p*-value < 0.05. The six macerated wines 1,2,3 originating from MC (red circle) and J (blue triangle) and analyzed in technical triplicates a, b, c. Red and blue ellipses correspond to the groups of macerated wines for MC and J, respectively with an interval of confidence of 95%. Loadings of the PCA are plotted in green and represent the eight molecular features associated with their RT (retention times) obtained in the chromatograms.

For volatile compounds, the differentiation of geographic sites in wines was less evident compared to powders as shown in Figure S1 and presented in the PCA in Figure 2. Only 8 molecular features associated with their RT (retention times) were differentiating origin with our statistical criteria and resulting in the separation of macerated wines based on gentian origin in the PCA presented in Figure 2.

In order to validate their ability to differentiate geographically the two gentian sites in the macerated wines, we work on extracted ions chromatograms specific to the major fragments of each molecular features in order to avoid possible coelutions with other CW wine volatile compounds in our interpretation. Only two of them were statistically still differentiating gentian origin in wine (Figure S3). The first volatile compound eluting at 15.79 min assigned to 2-methoxy-3-sec-butylpyrazine (RT-15.786) was only detected on JW. Already more abundant in Jura gentian roots compared to MC, its extraction in the Chardonnay wine medium enabled to preserve the gentian traceability in wine. The second compound was assigned to ethyl 9-decenoate (RT-21.191), it was absent from gentian roots analysis and originated from the control wine (CW). It has been described as a fermentative compound occurring during alcoholic fermentation and associated with floral notes in Chardonnay wines [31]. In the macerated wines, a statistical geographic distinction (*p* < 0.05) was still present, with a factor 2 lesser for MCW compared to JW. The statistical geographic distinction in macerated wine could be attributed to an ester adsorption mechanisms differing from MC and J gentian powders that could be attributed to a higher composition of biopolymeric absorbents [32], particularly polysaccharide composition for MC compared to J in the initial gentian roots (see Figure S3).

Such gentian root powder extraction process by Chardonnay wine reveals novel chemical markers that are not necessarily discriminating gentian origin in the powder’s analysis. The maceration process of gentian root powder in wine from different origins can lead potentially to two distinct beverages with typical sensory properties.

### 3.3. Sensory Characterization of the Wines

After transforming bitterness intensities into ranks for each panelist, the Friedman test gave a significant sample effect (Q = 46.03, *df* = 2, *p* < 0.0001). Post hoc Nemenyi test showed significant differences between CW and the two macerated wines, but not between MC and J (Table 3). Given the strong differences on bitter compounds showed in Table 2 between the two sites we expected significant sensory differences between MCW and JW. The reason of this surprising result could be that the levels of bitterness were so high that panelists reached saturation and could not physiologically discriminate the two samples in terms of bitterness. Furthermore, carryover effects during the tasting could have played a role in the panelists’ inability to discriminate between the two samples.

**Figure 3:**
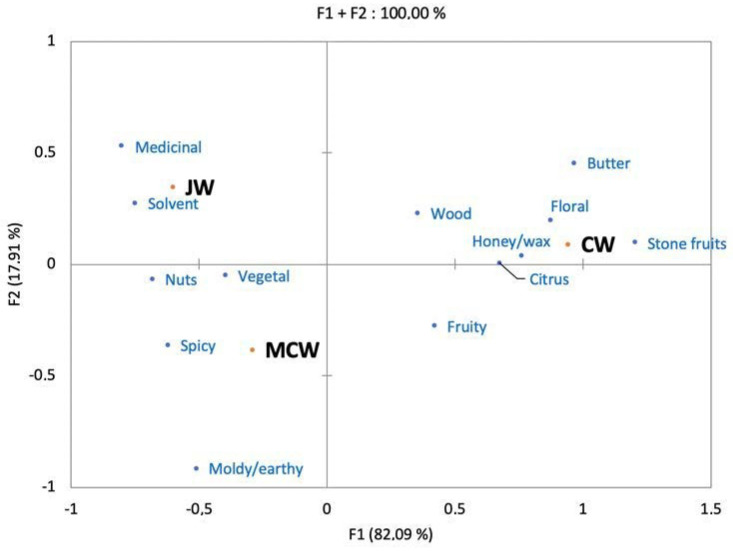
Correspondence analysis of the aroma description of the three wine samples.

Concerning the aroma assessment, Figure 3 shows the correspondence analysis (CA) of the frequencies of citation of each descriptor for each sample. Since we only have three samples the explained variance of the CA is logically 100% in only two dimensions. The first-dimension accounting for most of the explained variance opposes the control wine to the macerated wines. The results suggest that the fruity and floral notes associated with CW have been masked by aromas coming from the gentian powder. The second axis with only 17.9% of explained variance suggests that the two macerated wines are quite close in odor, but that JW could be slightly more solvent and medicinal while MCW would have a moldy/earthy nuance.

A deeper analysis of the differences between samples show slight, but significant differences between JW and MCW. A chi-squared test on the frequency distribution of fruity shows that MCW and CW are significantly fruitier than JW (*df* = 2; *p* = 0.0018). Moreover, chi-squared tests on the descriptors medicinal and moldy/earthy show also interesting results being medicinal significantly more cited for JW than for MCW (*df* = 1; *p* = 0.045) while MCW tend to be more moldy/earthy than JW (*df* = 1; *p* = 0.058).

## 4. Discussion of Chemically Differentiated Compounds between Two Gentian Sampling Sites and Sensory Properties in Macerated Wines

**Figure 4:**
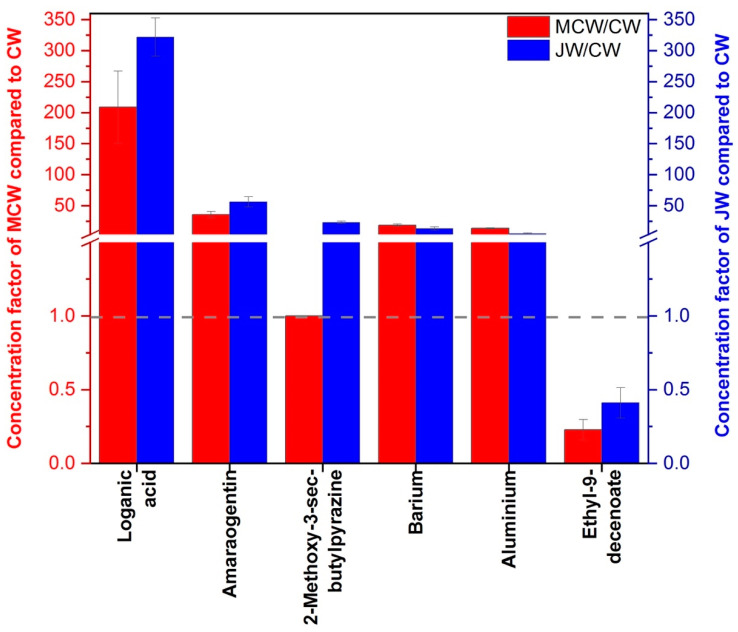
Concentration factor of chemical markers of gentian macerated wine that could explain sensory differences between macerated and unmacerated wine for MC and J gentian roots. The break on the y-axis was done from 1.5 to 3.9, minimum concentration factor value considered arbitrarily to affect sensory properties in macerated wine compared to CW. For loganic acid and amarogentin and 2-methoxy-3-sec-butylpyrazine, totally absent in CW, we consider for the calculation of concentration factor the concentration detection limit concentration or minimum area threshold values.

Figure 4 indicates the mean concentration factors of the validated (*p*-value < 0.05) chemical markers discriminating gentian geographic origin in the macerated wines (with *p*-value < 0.05) compared to unmacerated wine (CW). Only origin-differentiating compounds that could have a greater influence in sensory perception were chosen with a mean concentration factor above 3.9 or below 0.25 at minimum in one site for gentian- macerated wines. In this sense K, Ca and Sr were withdrawn from the discussion when applying this arbitrary criterium.

First of all, even if statistical differences in amarogentin and loganic acid concentrations were found in MC and J macerated wines (Table 2), and could have influenced the bitterness perception of the macerated wines, no difference was found in our experimentation surely attributed to the saturation of bitterness perception among wine tasters. Bitterness could have also been modulated by the mineral composition released in the macerated wines. Among them, cations like potassium and calcium are potential candidates, as previously proposed [12] but they were poorly extracted in the macerated wines. Their concentration factors range between 1 to 1.1 and between 1.5 and 2.1, respectively. In our study, barium increases by a factor comprised between 13 and 18 compared to unmacerated wine. This element has been described to suppress perceived intensities of sweetness, saltiness and bitterness and reduce palatability in beverages [33]. It should undoubtedly affect the sensory properties and act as a gentian geographic origin marker in the gentian macerated wines. The other origin-differentiating elements Sr and Al in macerated wines were concentrated between 1.7 and 3.8 and between 5 and 13.5, respectively. These two elements contribute poorly to sensory properties of wines, except aluminum that have been related to oxidation mechanisms by oxygen consumption in wines [34]. This could have occurred during wine maceration process with gentian leading to the observed changes of Lab coordinates (Table 1) with a decrease of the lightness (L) and an increase of the redness and yellowness component (a and b).

Among the volatile compounds, 2-methoxy-3-sec-butylpyrazine enables a geographic discrimination between Jura and Massif Central in both gentian roots and macerated wines. Its highest concentration in JW, compared to MCW, probably participates to the significantly higher medicinal notes conferred to the macerated wines. Unfortunately, our volatile data analysis in macerated wines did not bring forward robust statistical volatile markers of MC gentian that could be responsible of moldy/earthy notes in MCW. Nevertheless, compounds like trans-β-ocimene or 1,3,5,7-cyclooctatetraene, associated with herbal and moldy character, discriminating origin in the roots and no more discriminating origin in macerated wines could reflect lack of maceration time of MC gentian in Chardonnay wines or unveil synergetic effects in sensory descriptions of MCW. Additionally, fruity/floral notes, generally attributed to wine esters, were less present in macerated wines compared to unmacerated wine. Such sensory difference was evidenced chemically by ethyl 9-decenoate less presence in JW (factor 0.4) and in MCW (factor 0.2) compared to CW. This difference behavior in ester adsorption by gentian powder could have been responsible in the reduction of fruity/floral notes in macerated wines compared to CW (Figure 3). As the other main wine esters were not statistically different between JW and MCW, other sensory interactions should also participate in the observed decrease of fruity/floral notes in macerated wines. Pyrazines, like 2-isobutyl-3-methoxypyrazine, have been described to mask fruity characteristics and passion fruits attributes in Sauvignon blanc wines [35,36]. As any actual decrease of other wine esters was detected gentian pyrazines may mask fruity aromas perception in gentian macerated wines.

## 5. Conclusions

In this study, *Gentiana lutea* gentian roots powders originating from two sampling sites in France (Massif Central and Jura) were clearly distinguished by chemical volatile and non-volatile markers. During the maceration process of gentian roots in Chardonnay wines, gentian origin was still preserved in resulting wines with common and novel chemical markers affecting sensory properties of macerated wines. Among the common markers, calcium and 2-methoxy-3-sec-butylpyrazine enable to discriminate geographic origin in both gentian powder and macerated wines. Among the other discriminative geographic markers, chemical compounds originating from both gentian (amarogentin, loganic acid, Sr, Al, K, Ba) and ethyl-9-decenoate originating from Chardonnay wine should also be responsible of sensory differentiation in gentian macerated wines. Macerating gentian from two distinct origins in Chardonnay wine led to an overexpressed and undifferentiated bitterness perception and a global decrease of fruity/floral notes.

This study highlights: (i) that appropriate statistical processing of chemical data enables to validate chemical markers of gentian geographic origin in macerated wines differing from those in raw gentian roots powders due to differentiation in extractability and adsorption processes, (ii) bitterness in gentian macerated wines was not geographically discriminated due to a limited number of tasted macerated wines and an excessive bitterness perception, (iii) the sensory and geographic difference in gentian pyrazines and wine esters in gentian macerated wines should be deepened in future with quantitative measurements on wine volatiles.

Our findings would practically help in defining gentian maceration protocols taking in consideration an optimization of its chemical content to reveal bitterness and sensory differences in novel gentian-macerated alcoholic beverages.

## Figures and Tables

**Table 1:** Mean concentration (MC) of the three main bitter compounds analyzed and mineral elements statistically differing from the two sampling sites present in gentian roots from the two sampling sites. extracts, expressed in mg·L^−1^, with their associated mean standard deviation and the result of *p*-value from the analysis of variance between the two geographic sites. When the difference between sites is statistically different (*p*-value < 0.05) the symbol * is indicated on the right of the *p*-value.

Concentration (mg·L^−1^)	MC	J	*p*-Value
Gentiopicroside	724.69 +/− 79.46	700.24 +/− 130.25	0.795
Amarogentin	2.05 +/− 1.21	2.74 +/− 0.67	0.427
Loganic acid	98.45 +/− 43.89	106.13 +/− 31.76	0.819
Ca	1.03 +/− 0.60	7.30 +/− 4.21	0.003 *
Mg	21.80 +/− 12.59	8.00 +/− 4.62	0.03 *
K	11.45 +/− 5.22	5.51 +/− 0.66	0.122
Al	0.08 +/− 0.02	0.05 +/− 0.04	0.467
Ba	0.02 +/− 0.01	0.01 +/− 0.01	0.205
Sr	0.0012 +/− 0.0004	0.0013 +/− 0.0008	0.932

Values are mean of three replicates ± standard deviation. The symbol * indicates a significant difference (*p*-value < 0.05).

**Table 2:** Enological classical parameters and concentrations of main bitter compounds and mineral elements in gentian macerated wines from the two sampling sites.

	MCW	JW	*p*−Value
**Enological classical parameters**			
Ethanol (%)	12.05 +/− 0.10	12.13 +/− 0.03	0.357
Glucose/fructose	9.0 +/− 0.63	7.6 +/− 1.05	0.661
Total sugars (g·L^−1^)	15.67 +/− 0.73	14.3 +/− 1.21	0.877
Total acidity (g·L^−1^ H_2_SO_4_)	3.82 +/− 0.03	3.88 +/− 0.09	0.572
Volatile acidity (g·L^−1^ CH_3_CO_2_H)	0.16 +/− 0.02	0.15 +/− 0.03	0.501
pH	3.21 +/− 0.01	3.17 +/− 0.03	0.862
Malic acid (g·L^−1^)	0.37 +/− 0.06	0.3 +/− 0.06	0.518
Density	0.9955 +/− 0.0003	0.9952 +/− 0.0003	0.587
Color CIELAB L	72.85 +/− 1.32	71.87 +/− 2.25	0.432
a	18.10 +/− 0.39	18.73 +/− 3.32	0.191
b	91.35 +/− 1.69	92.52 +/− 4.36	0.237
**Bitter compounds (mg·L^−1^)**			
Gentiopicroside	134.42 +/− 97.23	447.49 +/− 221.95	0.089
Amarogentin	3.57 +/− 0.50	5.63 +/− 0.80	0.019 *
Loganic acid	20.87 +/− 5.81	32.16 +/− 3.09	0.041 *
**Mineral elements (mg·L^−1^)**			
K	682.48 +/− 21.80	637.05 +/− 8.00	0.028 *
Mg	108.33 +/− 5.03	102.67 +/− 3.79	0.194
Ca	84.46 +/− 1.03	114.39 +/− 7.30	0.003 *
Al	6.74 +/− 0.46	2.45 +/− 0.74	0.001 *
Ba	0.37 +/− 0.04	0.27 +/− 0.05	0.04 *
Sr	0.41 +/− 0.02	0.18 +/− 0.01	0.0001 *

Values are mean of three replicates ± standard deviation. The symbol * indicates a significant difference (*p*-value < 0.05). Values obtained for the unmacerated wine control wine (CW) are indicated in Table S1.

**Table 3:** Bitterness average ranks for each sample. Means with different letters were significantly different according to Nemenyi test (alpha = 5%).

Sample	Mean for Bitterness	Groups
JW	2.57	A
MCW	2.43	A
CW	1.00	B

## Supplementary Materials

The following are available online at https://www.mdpi.com/2304-8158/9/8/1061/s1, Table S1: Enological classical parameters and mineral constituents, expressed in mg·L^−1^ with their associated mean standard deviation, of unmacerated Chardonnay white wine CW. Figure S1: Hierarchical cluster analysis (HCA) realized on normalized volatile compound areas after validation of ANOVA and *p*-values < 0.05 (for all six aromatized wines 1, 2, 3 from originating from MC and J and analyzed in technical triplicates a, b, c). The color spans from green (minimum normalized area) to red (maximum normalized area) detected for each volatile compound after normalization of values for the entire dataset. Figure S2: Validated volatile compounds differentiating gentian geographic site in macerated wine, isolated from Figure S1 with mean area recalculated based on the extracted ion chromatogram of technical replicates a, b and c for each biologic macerated wine MCW and JW and unmacerated wine CW. Asterisk * represent a statistical difference between CW, MCW and JW based on an analysis of variance with *p*-value < 0.05. Figure S3: Polysaccharide content in gentian powders, expressed in equivalent of gentiobiose. Polysaccharide quantification was done using the phenol sulfuric assay using gentiobiose, supplied by Extrasynthèse, as the standard sugar compound. Letters indicate the statistical difference of the two groups MC and J by applying a Tukey’s honest significant difference post hoc test with a *p*-value < 0.05.
