# Supplementary material for: How Chemical and Sensorial Markers Reflect Gentian Geographic Origin in Chardonnay Wine Macerated with Gentiana lutea Roots?
Source: Foods. 2020 Aug 5;9(8):1061. doi: 10.3390/foods9081061 (PMC7466338; doi:10.3390/foods9081061)
Supplement: Supplementary file 1 [file foods-09-01061-s001.pdf]

foods

MDPI

Article

How chemical and sensorial markers reflect gentian geographical origin in chardonnay wine macerated with *Gentiana Lutea* roots?

Manon Biehlmann[1], Samvel Nazaryan[1], Emily Krauss[1], Mike Iron Ardeza[1], Stéphanie Flahaut[2], Gilles Figueredo[3], Jordi Ballester[4], Céline Lafarge[5], Elias Bou-Maroun[5], Christian Coelho[5,*]

[1] Master 2 Microbiology and Physicochemistry of Food and Wine Processes, Agrosup Dijon, Université de Bourgogne Franche Comté, 21000 Dijon, France ; manon.biehlmann@agrosupdijon.fr (M.B.); samvel.nazaryan@agrosupdijon.fr (S.N.); Emily.krauss@agrosupdijon.fr (E.K.); iron-mike.ardeza@agrosupdijon.fr (I-M.A.)

[2] CPPARM, ZA Les Quintrands, Route de Volx, 04100 Manosque, France ; stephanie.flahaut@cpparm.org (S.F.)

[3] LEXVA Analytique, 7 rue Henri Mondor, Biopole Clermont Limagne, 63360 Saint Beauzire, France ; g.figueredo@lexva-analytique.com (G.F.)

[4] Centre des Sciences du Goût et de l'Alimentation, AgroSup Dijon, CNRS, INRA, Univ. Bourgogne Franche-Comté, F-21000 Dijon, France ; jordi.ballester@u-bourgogne.fr (J.B.)

[5] Université Bourgogne Franche Comté, AgroSup Dijon, PAM UMR A 02.102 PAM, 21078 Dijon Cedex, France ; celine.lafarge@agrosupdijon.fr (C.L.); elias.bou-maroun@agrosupdijon.fr (E.B-M); christian.coelho@u-bourgogne.fr (C.C.)

* Correspondence: E-mail address: christian.coelho@u-bourgogne.fr; Tel: +33 3 80 39 61 95; Fax: +33 3 80 39 62 65.

Supplementary Materials:

Table S.1: Enological classical parameters and mineral constituents, expressed in $mg.L^{-1}$ with their associated mean standard deviation, of non-macerated Chardonnay white wine CW.

		CW
**Enological classical parameters**		
Ethanol (%)		12.25 +/- 0.10
Glucose/Fructose		0.5 +/- 0.2
Total sugars ($g.L^{-1}$)		3.9 +/- 0.1
Total acidity ($g.L^{-1}$ H_2SO_4)		3.56 +/- 0.03
Volatile acidity ($g.L^{-1}$ CH_3CO_2H)		0.08 +/- 0.02
pH		3.22 +/- 0.01
Malic acid ($g.L^{-1}$)		0.1 +/- 0.06
Density		0.9909 +/- 0.0003
Color CIELAB	L	90.71 +/- 1.32
	a	-1.2 +/- 0.39
	b	33.05 +/- 1.69
**Mineral elements ($mg.L^{-1}$)**		
K		641.0 +/- 0.6
Mg		80.4 +/- 0.02
Ca		54.50 +/- 0.01
Al		0.497 +/- 0.001
Ba		0.0228 +/- 0.0001
Sr		0.1070 +/- 0.0002

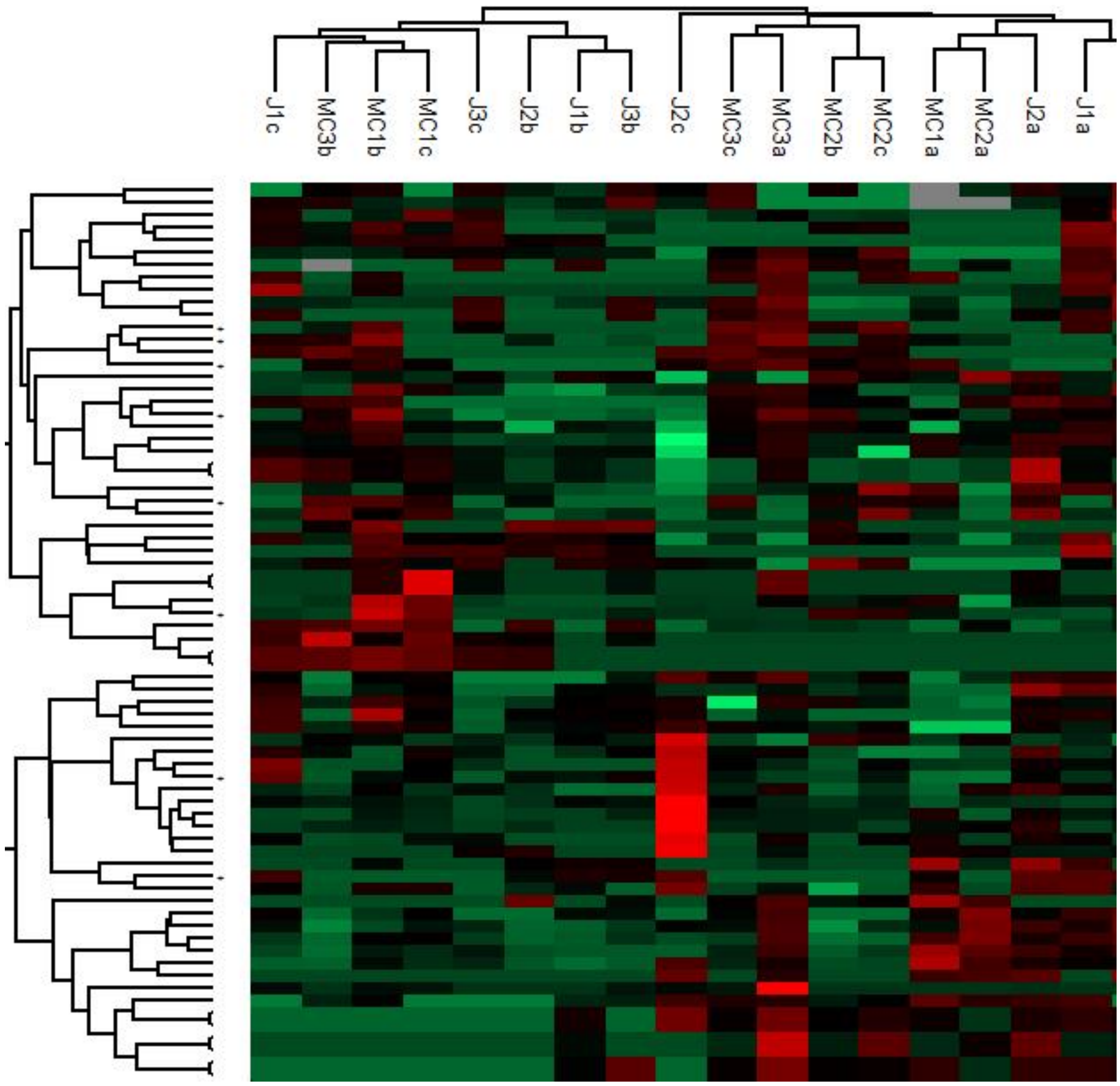

Figure S.1: Hierarchical cluster analysis (HCA) performed on normalized volatile compound areas after validation of ANOVA and p-values < 0.05 (for all six aromatized wines 1,2, 3 from originating from MC and J and analyzed in technical triplicates a, b, c). The color spans from green (minimum normalized area) to red (maximum normalized area) detected for each volatile compound after normalization of values for the entire dataset.

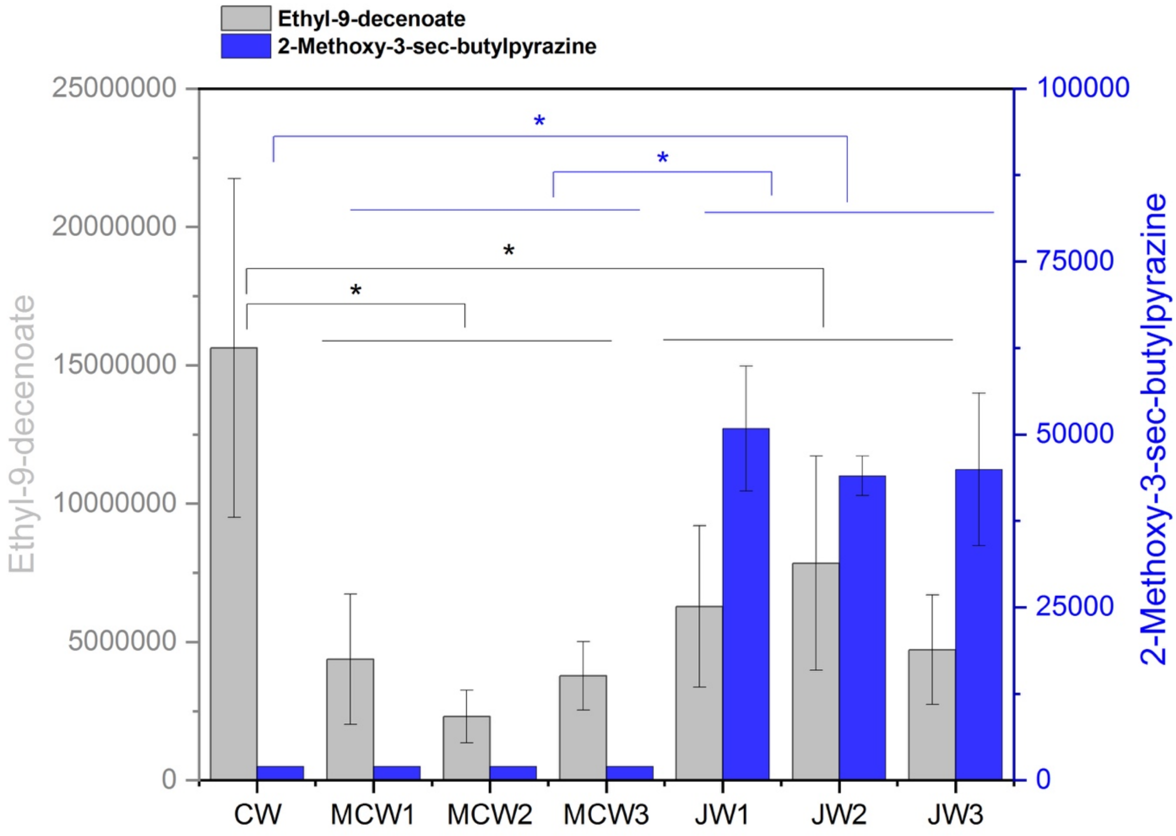

Figure S.2: Validated volatile compounds differentiating gentian geographical site in macerated wine, isolated from Figure S.I.1 with mean area recalculated based on the extracted ion chromatogram of technical replicates a,b and c for each biological macerated wine MCW and JW and non-macerated wine CW. Asterisk * represent a statistical difference between CW, MCW and JW based on an analysis of variance with p-value < 0.05.

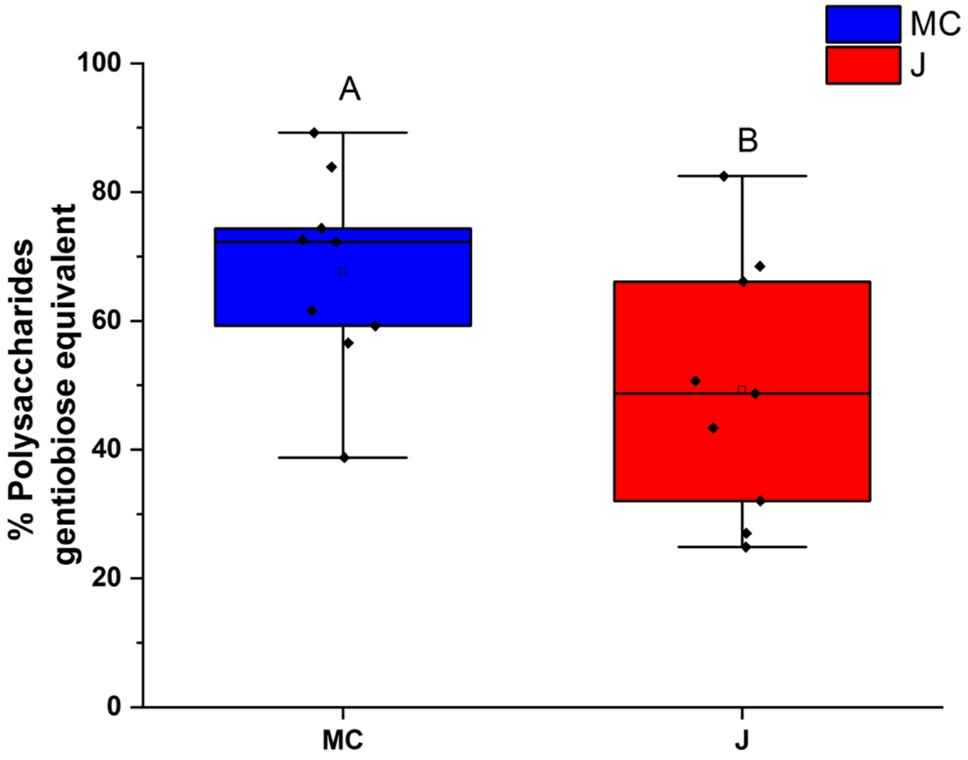

Figure S.3: Polysaccharide content in gentian powders, expressed in equivalent of gentiobiose. Polysaccharide quantification was done using the phenol sulfuric assay[1] using gentiobiose, supplied by Extrasynthèse, as the standard sugar compound. Letters indicate the statistical difference of the two groups MC and J by applying a Tukey's Honest Significant Difference post hoc test with a p-value < 0.05.

[1] Usseglio-Tomasset, L., Castino, M. (1975). I colloidi solubili di natura glucidica dei mosti e dei vini. Parte I. Riv. Viticolt. Enol., 28, 374-391.
